# Projected effects of ocean warming on an iconic pelagic fish and its fishery

**DOI:** 10.1038/s41598-021-88171-1

**Published:** 2021-04-22

**Authors:** Vicenç Moltó, Miquel Palmer, Andrés Ospina-Álvarez, Sílvia Pérez-Mayol, Amina Besbes Benseddik, Mark Gatt, Beatriz Morales-Nin, Francisco Alemany, Ignacio A. Catalán

**Affiliations:** 1 IMEDEA, CSIC/UIB, Miquel Marques 21, 07190 Esporles, Illes Balears Spain; 2 Instituto Español de Oceanografía, Centre Oceanogràfic de Balears, Moll de Ponent S/N, 07015 Palma de Mallorca, Spain; 3 INSTM (Centre of Monastir), Road of Khniss 5000, Monastir, Tunisia; 4 Department of Fisheries and Aquaculture, Ministry for the Environment, Sustainable Development and Climate Change, Fort San Lucjan, Triq il-Qajjenza, Malta

**Keywords:** Climate sciences, Ecology, Environmental sciences, Ocean sciences

## Abstract

Increasing sea temperature is a driver of change for many fish traits, particularly for fast-growing epipelagic species with short life spans. With warming, altered spawning phenology and faster growth may produce substantially larger body sizes of the new cohort, affecting fishery productivity. We present an individual-based model (IBM) that predicts the distribution of fish length at catch under observed and projected thermal scenarios, accounting for mortality, temperature-dependent spawning phenology, temperature- and photoperiod- dependent growth. This IBM was demonstrated with *Coryphaena hippurus* (common dolphinfish), a circumglobally-distributed and highly thermophilic species sustaining commercial and recreational fisheries where it is present. The model projected a 13.2% increase in the average length at catch under marine heatwave conditions compared to the current thermal regime (1995–2005 average). Projections under RCP scenarios 4.5 and 8.5 by the end of the century led to 5.1% and 12.8% increase in average length, respectively. Furthermore, these thermal scenarios affected spawning phenology differently, producing higher variance in body size under RCP 8.5 scenario with respect to marine heatwave conditions. This study highlights how the environmental effects of climate change can alter the distribution of species length at catch.

## Introduction

Global environmental change is having measurable effects on the epipelagic realm in marine systems. Documented impacts include increases in sea surface temperature (SST), changes in water mass circulation and mixed layer depths, altered primary productivity, or ocean acidification^1–3^. Importantly, the effects of climate change are also driving an increase in the frequency and intensity of marine heatwaves (MHWs)^4,5^. These effects, either directly or through trophic interactions, impact the species inhabiting the epipelagic environment, including those that support high commercial value fisheries, such as tunas or forage fishes^6,7^.

The common dolphinfish, or mahi-mahi (*Coryphaena hippurus*, Linnaeus 1758), is a good candidate species for understanding the effect of warming on individual and population traits. Dolphinfish is a large pelagic species inhabiting the surface layer of the global tropical and subtropical oceans. It exhibits a voracious feeding behavior, required to sustain one of the highest metabolic and growth rates recorded for teleostean fish^8–10^. It can reach approximately 20 cm in its first month and 150 cm after its first year of life within a reduced life-span of up to 4 years^9,10^. Furthermore, this species constitutes an important global fishing resource. The commercial captures have globally increased over the last decades from 7,103 t in 1950 to 115,658 t in 2014^11^, and it is also a valued target species for sport fisheries^10^.

The Mediterranean Sea encompasses the northern limit of the Atlantic regional distribution of dolphinfish and supports important recreational and commercial small-scale fisheries based on age-0 individuals^10^. Essentially, the juveniles (approx. 20–60 cm in length) are fished around fish-attracting devices (FADs) between September and December^12^. These FADs account for approximately 30% of all FADs deployed worldwide^13^. This region is particularly suitable for analyzing the effects of warming on growth and fisheries since it has been defined as a hotspot for climate change^14,15^. The SST projections under the intergovernmental panel on climate change (IPCC) representative concentration pathway (RCP) 8.5 scenarios for the end of the century suggest that the average SST may increase by approximately 3 °C over current climatological values^16^. In addition, simulations from coupled regional climate system models project at least one long-lasting MHW every year, which may last up to three months longer and be approximately four times more intense and 42 times more severe than present-day events^17^.

Changes in environmental conditions in the epipelagic zone could rapidly induce observable changes in the physiological and ecological traits of fish species, including growth^18,19^. Several studies have addressed the growth patterns of dolphinfish, mainly through examining calcified structures (otoliths and scales), both in the Mediterranean Sea^10^ and other regions of the global ocean ^10,20^. The results show that the growth pattern of this species differs between regions, even within the Mediterranean Sea^10,20^. The origin of these growth differences can be attributed to several potential causes, including genetics, age-estimation biases, or environmental drivers, and there is an identified knowledge gap on the potential effect of environmental factors on the growth patterns of this species and its productivity^21^.

**Figure 1:**
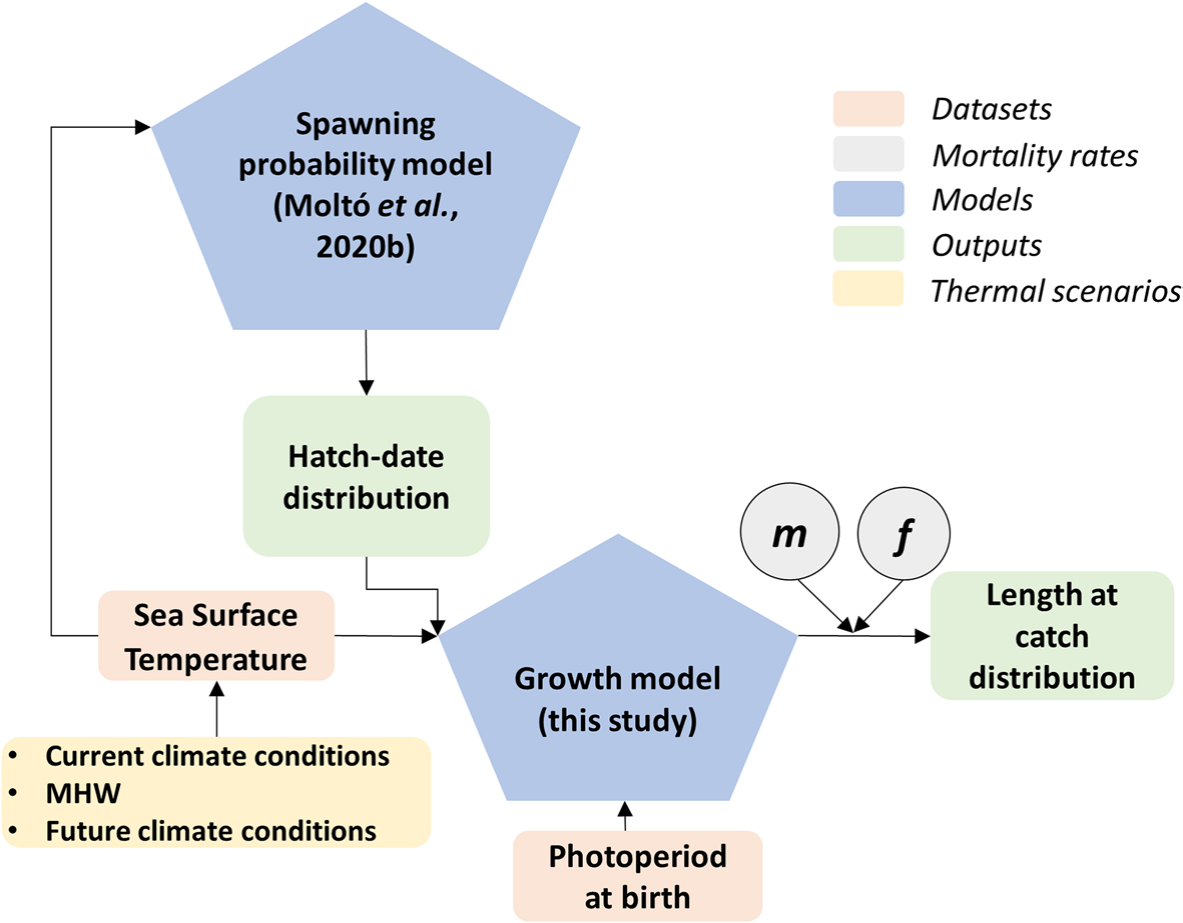
Conceptual scheme of the IBM used to project the fish size distributions under future thermal scenarios and an observed MHW, considering ecological processes and fishery drivers. Created with Microsoft PowerPoint, 2011 (https://www.microsoft.com/es-es/microsoft-365/powerpoint).

This study developed an individual-based model (IBM) for age-0 dolphinfish to resolve how the environment, namely temperature (current, referring to the 1995–2005 average, and projected using RCP 4.5 and RCP 8.5 scenarios for 2080–2099) through life history and photoperiod at birth affects the length distribution of the potential catch. The IBM (schematized in Fig. 1) was initialized by a spawning probability model that relates the temperature to the spawning time to accommodate potential spawning shifts under varying thermal regimes^22^. In a second step, a biochronology-based temperature-dependent growth model was used, which combines two growth sub-models. The first growth sub-model relates otolith radius-at-age with age and accumulated temperature (individual thermal history) to predict the otolith radius at catch. The second growth sub-model estimates the fish length from the otolith radius at catch, temperature at birth and photoperiod at birth (as a proxy for feeding time)^23^. The IBM also incorporates the effects of both natural and fishing mortality when predicting the length distribution at catch at the population level. This phenomenological model is applied forced with different thermal scenarios, projections of surface ocean warming and a MHW, providing an understanding of the effects of environmental conditions on the spawning phenology and growth of dolphinfish. This type of model does not require extensive data, can be potentially applied to other similar species and the resulting information can be useful to fishery managers in the context of adaptation to global warming.

## Results

**Figure 2:**
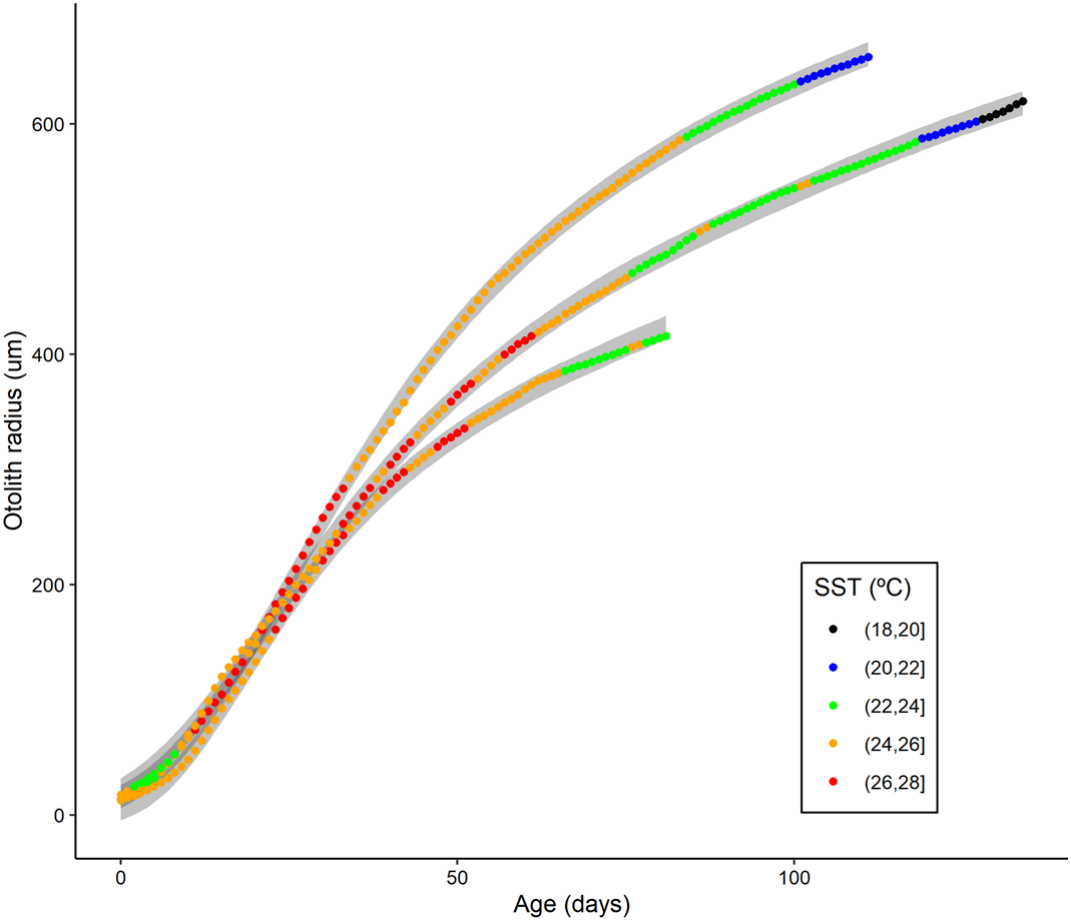
Adjustment of the temperature-dependent Gompertz growth model to three individuals selected for their contrasting characteristics of otolith growth, age, and thermal history. The observed values for each individual are presented as colored circles, where the color represents the temperature range estimated from satellite data. The confidence bands are plotted at 0.90 of the confidence interval for clarity. Graph created with R 3.6.2 (https://www.r-project.org/)^24^ using the ggplot2 package^25^.

The growth model developed here, composed of two sub-models, has proven to be a useful approach to analyze environmental dependencies on fish growth. The first sub-model consists of a temperature-dependent Gompertz model that predicts the otolith radius-at-age at a daily scale. The thermal effect is incorporated as the accumulated temperature experienced each day and related linearly to the individual *L∞* parameter, accounting for age (see Methods section). This sub-model was calibrated with 1,894 observations of otolith daily increments from 20 individuals captured in different Mediterranean regions that had experienced different temperature regimes prior to capture (see Supplementary Information). This sub-model was able to predict the observed otolith growth patterns within a confidence interval between 0.93 and 0.95 (Fig. 2).

**Figure 3:**
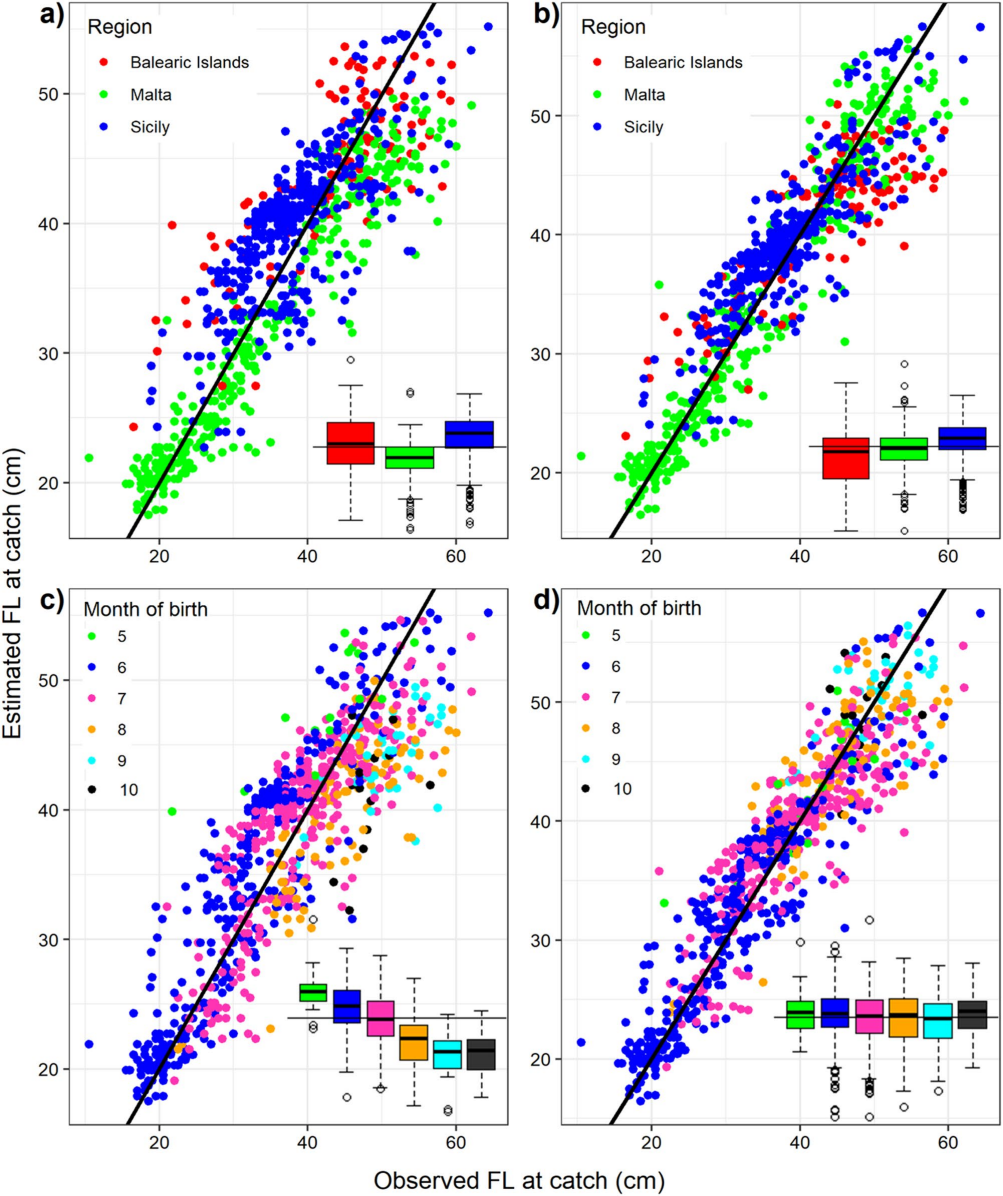
Estimated vs. observed fish length (furcal length, FL) at capture of the 876 individuals used for model validation. Two versions of the model are represented. A first version (panels **a**,**c**) corresponds to the environmental-independent model (not accounting for temperature and photoperiod at birth). A second version (panels **b,d**) corresponds to the environmental-dependent model with the best DIC. The boxplots in each panel show the residuals’ distribution by region (**a**,**b**) and month of birth (**c**,**d**). The environmental-dependent model version largely corrects the biases, which can be seen as a better horizontal alignment of the boxplots, therefore not over or underestimating the length at catch. Graph created with R 3.6.2 (https://www.r-project.org/)^24^ using the ggplot2 package^25^.

The second growth sub-model can predict the fish length-at-catch from the otolith radius at-catch (which has been estimated with the first sub-model) and the temperature and photoperiod at birth (See “Methods”, Assumption 2). This second sub-model was calibrated and validated using a dataset containing 1876 aged individuals (different from those in the first sub-model) of known length from different Mediterranean locations. The otolith radius of these individuals was estimated using the first growth sub-model. Subsequently, 1000 randomly selected individuals were used to estimate the parameters of this second sub-model and the remaining 876 were used to validate the length estimations (Fig. 3). Figure 3 shows the estimated vs. observed values of fish length at capture. The environment-dependent model clearly improved temporal and spatial biases compared to a model run without environmental dependencies (Fig. 3a,c). All parameters were optimized on an integral growth model (the two growth sub-models combined), adopting a Monte Carlo approach with uninformative priors for the unknown parameters (see Methods section). This integral growth model results from the best parameters combination (i.e., two lags to avoid autocorrelation in the first sub-model and the temperature and photoperiod at birth as predictive variables in the second sub-model. See model technicalities in the Supplementary Information), better predictive error in terms of deviance information criterion (DIC), and better accuracy in terms of root-mean-square deviation for the validation dataset (4.06 cm versus 5.10 cm for the model without environmental parameters). Precision (measured as the mean interquartile range) of the length at catch estimates were comparable but slightly wider for the environmental-dependent model (0.7 cm vs 0.3 cm). The potential scale reduction statistics (R hat) for all the parameters of the environmental-dependent model were equal or less than 1.01, which strongly suggests a good convergence. The estimated parameter values are provided in the Table S1 of the Supplementary Information.

**Figure 4:**
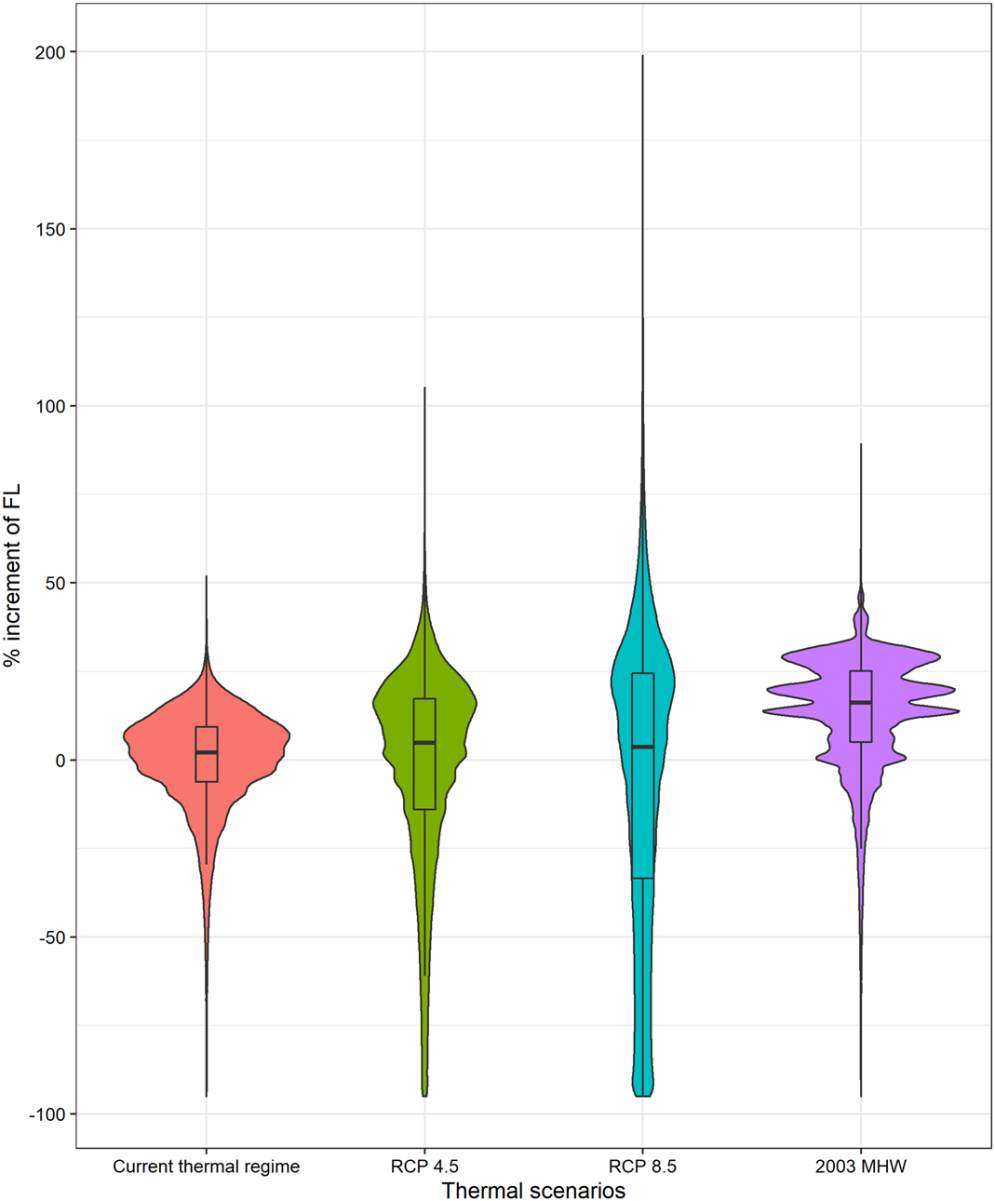
Violin plots (probability density) showing the percentage of increase in fish length (FL) with respect to the average fish length at catch under the current thermal regime (average 1995–2005) for projected thermal scenarios (RCP 4.5 and RCP 8.5 for 2080–2099) and the 2003 MHW. The evaluation date of the length distributions was the 15th of September in all cases. The boxplots represent the interquartile range and the horizontal black line corresponds to the median value of the correspondent distribution. Graph created with R 3.6.2 (https://www.r-project.org/)^24^ using the ggplot2 package^25^.

In summary, the growth model developed here outperformed simpler models, and can predict the fish length at capture with reasonable accuracy and precision. This model was incorporated into an IBM (Fig. 1) to project the length at catch distribution forced with future temperature regimes from RCP scenarios and a MHW. This IBM not only accounted for the environment-dependent growth but also considered a temperature-dependent spawning probability as well as mortality processes. The model projected the mean length of the individuals incorporated into the fishery (from 20 cm FL onward) at any given time. As an example, we calculated the length distribution one month after the legal opening of the fishing season. This length is expected to increase by 5.1% and 12.8% under the RCP 4.5 and RCP 8.5 scenarios, respectively (Fig. 4). In the case of the simulated MHW, we used the well-known MHW recorded in 2003 in the Mediterranean (see Methods). During this episode, the mean length increased by 13.2%. There was also a noticeable change in the shape of the expected length distribution. Whereas a relatively high number of smaller-than-average individuals are projected in future scenarios, this was not the case for the MHW simulation, which yielded a length distribution shaped similarly to that of the current thermal regime but with larger individuals.

## Discussion

Biochronologies provide the capacity to analyze how environmental drivers affect growth, rebuilding the past environmental effects on calcified structures, allowing us to infer the effects of environmental variability of growth^26^. Here, we take advantage of the environmental footprints on fish otoliths to develop a temperature-dependent growth model. This model has been included in an IBM that enables a better understanding of how thermal variability may affect dolphinfish size at catch in the NW Mediterranean.

We successfully reproduced a large portion of the variability in dolphinfish growth. Additionally, we provide an example of using this model in the short- and long-term projection of fish size, under future thermal scenarios and extreme events. Such projections are informative to anticipate potential consequences for the fisheries.

The assumption that the SST is a key driver of growth was confirmed. Similar results have been reported by modeling the growth from the daily otolith increment widths in small pelagic fish (*Engraulis encrasicolus* and *Sardina pilchardus*) in the same Mediterranean Sea basin^27,28^. In both studies, the authors argued that the temperature significantly affects growth and explains a large proportion of the variance in daily growth rates within an optimum temperature range. A similar strong temperature effect has been observed for the European anchovy^29^ and the Atlantic horse mackerel^30^ in the adjacent Atlantic region, as well as for other pelagic species in other regions of the world^31^.

The photoperiod acts as a spawning trigger that is crucial for allowing an individual to attain a determinate length to survive, given other relevant environmental conditions (temperature, food availability) stay within adequate (but maybe suboptimal) limits^32^. If temperature and food availability can sustain positive growth, longer days will allow greater foraging capacity and increased growth^23,33^. In the case of the dolphinfish, which are visual predators with high energy demands, this is relevant. Consequently, a larger time-availability to feed at earlier stages (around the summer solstice) did correct growth-related biases in the predicted fish length at catch, especially those attributed to the month of birth. Furthermore, the individual and between-regions growth differences were already determined at early stages^34^. As the dataset to implement the growth model was based only on the “survivors”, we can assume that underperforming individuals were eliminated from the population, and only high-performance individuals were examined. Although some effect of feeding into growth (through photoperiod) is accounted for at the earliest stages, we recognize that there is no reliable information to explicitly incorporate the effect of food availability throughout the life span of fish. The effect of the variability of feeding on growth based on otolith readings can be masked by factors such as growth inertia, potential selection through ontogeny, and otolith accretion due to the buffer effect of reserves, especially in larger individuals^35^. Potentially, the effect of food could be better incorporated within a bioenergetics context^35–37^.

The use of future thermal scenarios in this study was only exploratory, and several considerations should be made regarding the limitations of the simulations. On the one hand, statistical methods based on observations may have limited value in long-term forecasting if systems are expected to depart largely from their current conditions^38^. These departures may include significant changes in prey or predators, which could substantially modify mortality estimates. On the other hand, projecting ecosystem changes in the Mediterranean may be particularly problematic because, for example, the biogeochemical models required for complex projections cannot even consistently reproduce inter-annual variability^39,40^.

Several studies assessed environmental effects on fish growth but few have projected these effects in future climate scenarios^41^. In the case of the Mediterranean, the projection of complex models to analyze changes in growth or distribution (e.g., multispecies models coupled to physical-biogeochemical outputs) has only just begun, and the relatively simple models of thermal effects on fish growth tend not to incorporate potential phenologic changes or the different sources of mortality. Existing models that project, among other variables, fish growth, show that projections are only useful at relatively high spatial aggregated scales^39^. For juvenile dolphinfish, this is particularly valid because forcing variables are surface temperature and photoperiod, and the species shows relatively high mobility.

Our growth projections under warmer scenarios showed that significant increases in fishery-available sizes are expected. An interesting feature of the projections was that the future thermal scenarios increased the spread of sizes and included a higher abundance of small specimens than the MHW simulation. The model used to determine the probability of birth as a function of temperature was parameterized with the gonadosomatic index (GSI) from different populations of different regions worldwide^42^. In tropical regions, the spawning pattern of the population is less intense but continuous throughout the year, while at higher latitudes, spawning is more synchronized within the few hotter months^10^, and these dynamics were well captured by the spawning component of the model^42^. Because the future climate scenarios (especially RCP 8.5) show temperature annual cycles that are more similar to those in tropical regions (although some seasonality is still clear), the model projects spawning dynamics more similar to those in tropical conditions, which will result in a higher likelihood of finding individuals in a broader range of sizes during the fishing season (Fig. 4). In contrast, this effect was not reproduced in the case of the 2003 MHW because the seasonal temperature dynamics were similar to the current ones but with higher temperature values during a relatively short period (Figure S3, Supplementary Information). Thus, the spawning phenology in the 2003 MHW was almost unaffected, but the growth rates were higher than in current conditions.

Experimental and field data support that within physiologically tolerable thermal ranges, an increment in temperature causes an increase in the metabolic rate, with a consequent increment in fish growth rates^27,41,43,44^. However, if the temperature exceeds or decreases over the tolerance limits (species and stage-specific), the growth rate will be negatively affected^31,45,46^. The thermal effects will interact with other biotic^47^ or abiotic factors^45^. Our IBM does not explicitly account for these complex effects. However, it indirectly accounts for thermal tolerance levels, for the early stages, through the temperature-dependent spawning sub-model (Fig. 1). As mentioned above, this species inhabits all tropical regions in the world, where maximum temperatures are similar to those predicted for RCP 8.5 in the Mediterranean, sometimes reaching 33 °C (see Supplementary Information). Therefore, we contend that these thermal projections would not negatively affect growth, even within the framework of theories considering adverse effects of temperature on ventilation^46^, as long as food availability is sufficient to maintain the increased metabolic rates.

Our model does not incorporate some important processes that may alter the length distribution of future catch at a given point in time. For example, the model is not currently connected to potential effects of the stock size (e.g., via density-dependent effects) on growth. In general, our model, and most of the statistical models projecting thermal effects lack consideration of plastic/genetic adaptations of the species to long-term changes, which cannot be discarded. However, some thermal effects related to global warming can occur suddenly with little chance for adaptation (e.g., MHWs^4^), and our model can help to predict some of its consequences. Unfortunately, we could not validate the effects of the 2003 MHW due to lack of data. It is known, however, that this MHW had a strong impact on the early stages of the Atlantic bluefin tuna (*T. thynnus*) in the area, increasing their survival, usually related to higher growth rates, and subsequent cohort strength^48^. The consideration of future thermal effects on stocks will need to incorporate these potential effects on the early stages, which under experimental conditions acted as bottlenecks under climate change scenarios^18,49^.

Our study accounted for ecological processes (spawning window shift, daily growth, mortality processes) and fishery drivers (mortality, size availability, and fishing season constraints) that have not been considered before for the species. All this information was translated into a population-based simulation focusing on the potential consequences for the average catch size within a framework of global warming. As mentioned above, the stock is not assessed in this region and FAO has recently called for the need to better understand its population dynamics^10,50^. This work demonstrates that provided the annual thermal field (through satellites, predictive models or projections), the threshold values for spawning can be inferred, and then, estimates of growth and mean size at any catch date can be derived accounting for simple mortality rules. Our results may be relevant in the context of fishery management. For example, the legal start of the fishing season for Mediterranean European countries is fixed, roughly coinciding with the time at which juveniles attain marketable body size^10^. This timing may shift in the face of global warming and MHWs, through accelerated growth and/or changes in spawning phenology. Our model can be used to analyze these shifts at different time scales, given information on surface temperature and mortality proxies are available. As discussed above, the model has limitations. Therefore, the application of our results would benefit from comparative exercises with other fishery models, once they become available^51^.

## Methods

We describe below the underlying assumptions and hypotheses, the environmental data sources, and the IBM components grouped in two main sections: the temperature-dependent spawning model and the growth model. Finally, we describe the simulated scenarios.

### Underlying assumptions and hypotheses

A1: In temperate areas, where SST can change quickly, differences of a few weeks in hatch dates will have, on average, more thermal impact on growth than being located a few hundred kilometers apart at similar latitudes. In the Mediterranean, dolphinfish spawn after the onset of summer stratification^52–54^. This period is associated with the high thermal stability of the epipelagic area and a well-defined mixed layer^55,56^ where this species occurs^57,58^. After 1.5–4 months, juveniles are captured at the FADs around the coast, where they approach to feed^53,57^. Therefore, for a given summer day in this area, the surface temperatures are relatively homogeneous in large regions. Therefore, we assumed that the daily average thermal conditions for an age-0 individual could be approximated by the average SST inside the polygons encompassing the four regions considered in this study (Balearic Islands, Malta, Sicily and Tunisia). The average area of the polygons ranges from approximately 12,000 to 19,800 squared nautical miles (Figure S1, Supplementary Information), covering from oceanic to coastal areas where the FADs are located and larval records exist^10^. The few animals from Tunisia were only used to derive extreme temperatures for the growth sub-model, whereas the other three areas were used for the full model.

A2: The physical-biological constraints during the planktonic stages of larval fish are crucial to determine the future growth trajectories, and they must be specifically considered. Several studies have shown that individual differences in growth seem to be determined at early stages, and fast-growing species set their growth trajectory particularly early^30,34^. Therefore, we explicitly considered the temperature at birth as a key parameter in the model that accounted for the thermally related individual-based differences in the establishment of growth rates. The surface production of Mediterranean offshore areas during the dolphinfish spawning season is low^59^, and the trophic ecology of the larval stages of this species in the Mediterranean is unknown. We assumed that the feeding intensity during the larval phase is partly dependent on daylight hours^23,33^, and we used the photoperiod at birth as a proxy for the potential larval feeding time. After this critical life stage, and given the extremely fast-growth and broad trophic niche that juvenile dolphinfish exploit^10^, we also assumed that there was no food limitation for individual fish.

Under the latter assumptions, we hypothesized that the observed among-individual differences in juvenile growth trajectories should be consistently related to the different individual thermal histories modulated by the conditions experienced in the early life stages. Therefore, a temperature-dependent growth model that considers photoperiod as a proxy for food availability in the early life stages could be built from otoliths collected from age-0 individuals born (and growing) under diverging thermal constraints, either early or late in the season within a determinate region or on similar dates but in different regions. We integrated the individual growth model predictions developed in this study, together with other key concurrent effects on size distributions, such as temperature-mediated spawning phenology and mortality processes, in an IBM to project the potential shift of the size distribution at a population level (Fig. 1).

### Environmental data

The main forcing variable of the model is the thermal history of each individual (see below for details on samples). This information was used to calibrate and validate the growth model. The mean daily SST value of each region where larvae and juveniles were assumed to occur (Figure S1, Supplementary Information and assumptions) was assigned for each day of life based on the capture date and age of each individual. The daily SST experienced by the sampled fish was obtained at a 4 km resolution for all years with otolith observations using the Copernicus Marine Environmental Monitoring Service (CMEMS, prod. “SST_MED_SST_L4_REP_OBSERVATIONS_010_021_a”). The photoperiod was obtained from the solar calculation resources from the NOAA Earth System Research Laboratory, Global Monitoring Division (https://www.esrl.noaa.gov/gmd/grad/solcalc/calcdetails.html).

The surface thermal projections of warming scenarios to perform the simulations under future environmental conditions were based on a regionalized oceanographic (climatic) model corrected for annual temperature biases using average satellite observations. Further details of this model and its implementation are provided in the Supplementary Information. The thermal projections were elaborated according to the IPCC RCP 4.5 and RCP 8.5, and the current climate conditions were assumed to be the 1995–2005 average because this period included the vast majority of the available otolith/length data used in our empirical model, and because the thermal scenarios from RCP 4.5 and RCP 8.5 were almost identical for this current period. The thermal projections were run for the last two decades of the century (2080–2099) at a weekly resolution. This period was chosen to facilitate comparisons with several projections relevant to fisheries^60^.

We also simulated the conditions of a marine heatwave as an example of an extreme thermal event with a magnitude and duration that can severely affect biological processes and that is expected to increase with climate change^4,5^. As a case study, we used the well-described 2003 MHW in the study region^61,62^, with demonstrated effects on biological systems^48,63^. This MHW yielded a temperature of 2–3 °C over the average in the Mediterranean and lasted over a month. According to the standardized classification, this MHW was categorized as “Category II, Strong” ^64,65^, which was confirmed in our subarea of analysis (Marine Heatwave Tracker platform^66^). The thermal regime for our 2003 MHW simulation was obtained from satellite data over the selected case study area (Figure S3, Supplementary Information).

### Temperature-dependent spawning probability

Projecting growth changes in a population under an environmental change framework must also incorporate the potential consequences of phenological changes into the spawning dynamics^22^. For this reason, a temperature-dependent spawning probability model developed by the authors^42^ (Fig. 1) was used as the input to initialize the IBM. This model is empirically based on the hatch-date distribution inferred from otolith readings but corrected for the mortality effects on that inference and taking into account the relationships between the temperature and gonadosomatic indices (GSI) for this species around the world^10^. Here, we use this model to generate a probability density function of hatching dates as a function of temperature.

### Growth model

The phenomenological model used in this section was composed of two sub-models. For the first sub-model (Eqs. 1 and 2), the Gompertz growth model^67^, which appropriately describes juvenile dolphinfish growth^68^, was used to fit the otolith daily growth increments from each individual of the otolith readings dataset (see Supplementary Information) as follows:

1 $${OtR}_{\left(i,age\right)}={L}_{\infty \left(i,age\right)}{e}^{-{b}_{\left(i\right)}^{{e}^{\left(-{c}_{\left(i\right)}*age\right)}}}$$

where *OtR*_*(i,age)*_ is the expected otolith radius at *age* (for each day of life) of the *i* fish and *L*_*∞,(i,age)*_, and *b*_*(i)*_ and *c*_*(i)*_ are the Gompertz growth parameters for each individual. Note that *L*_*∞,(i,age)*_ is not only fish-specific but also varies with age to account for the specific thermal history experienced by each *i* fish. The temperature effect is incorporated through the accumulated temperature (sum of the average temperature at 00:00 h of each day) experienced by the individual along with its life history over the *L*_*∞*_ growth parameter considering a linear relationship since similar dependencies in growth model parameters have been suggested^69^:

2 $${L}_{\infty \left(i,age\right)}={L}_{\infty ,0\left(i\right)}+{L}_{\infty ,slope}{CummTemperature}_{age}$$

where *L*_*∞,0(i)*_ is the intercept parameter for each *i* fish, *L∞,slope* is the regression slope and *CummTemperature*_*age*_ is the accumulated temperature from birth to *age*.

Given that within-fish radius-at-age residuals were temporally autocorrelated (see model technicalities provided as Supplementary Information), *n* additional terms were added to the expected value from Eq. (1). Each of the *n* terms added to a given radius-at-age was proportional to the residual (observed minus expected from Eq. 1) of the preceding 1 to *n* days. The number of lags considered (*n*) was increased one by one until the residuals did not show temporal autocorrelation (2 lags, see Supplementary Information). The confidence interval of the otolith radius estimates was obtained from intervals of 50 days from a confusion matrix calculated with the confusionMatrix function from the R package *caret*^70^.

The otolith radius is closely related to dolphinfish length and can act as a proxy for somatic growth^68^. Accordingly, the objective of the second sub-model (Eq. 3) was to estimate the fish length once the otolith radius had been estimated for a given fish. For this, the first sub-model (Eqs. 1 and 2) was used to predict the expected otolith radius at the capture day for the individuals of the calibration/validation datasets (1876 age-0 individuals for which the fish length at catch, age, capture date and spatial origin were known, see additional details in the Supplementary Information).

The fish length (FL) at catch was assumed to depend on the estimated otolith radius, the temperature at birth, and the photoperiod at birth (see Assumption 2), as follows:

3 $${FL}_{\left(i\right)}={OtR}_{(i)}\left({\beta }_{0}+{\beta }_{T}{T}_{b\left(i\right)}+{\beta }_{P}{P}_{b\left(i\right)}\right)$$

where *FL*_*(i)*_ is the fish length at catch for each *i* fish, *OtR* stands for the otolith radius at catch (estimated from Eqs. 1 and 2), *β*_*0*_, *β*_*T*_, *β*_*P*_ are the regression parameters, *T*_*b(i)*_ is the temperature at birth and *P*_*b(i)*_ is the photoperiod at birth experienced for each individual.

The parameters of the integral model (i.e., combining Eqs. 1, 2 and 3) were fitted using a Bayesian approach. The samples from the joint posterior distribution for the fish length of the validation dataset individuals and the other model parameters given the data (otolith readings dataset and calibration dataset) were obtained using JAGS and R2jags packages^71,72^ of the R statistical software version 3.6.2^24^. Virtually uninformative priors were used for all unknown parameters. The input data and additional details on the prior settings and other technicalities of the model structure are available as R files and scripts, respectively, and are provided as Supplementary Information. Three Monte Carlo Markov chains (MCMC) were run, and their convergence was assessed visually using the Gelman-Rubin statistic. The posterior distributions of the model parameters were estimated from 100,000 valid iterations after appropriate burning (the first 2000 iterations were discarded) and thinning (only one out 10 iterations were kept).

The integral model was calibrated (parameter estimation) using all fish from the otolith reading dataset and the calibration fish dataset (1000 individuals from which the furcal length, region and age were known). The remaining 876 individuals with the same information were used to validate the model (see additional details in the Supplementary Information), determining the model precision (mean inter-quartile range) and accuracy (square root of the mean squared differences between the observed and the predicted fish length at catch).

### Simulations of the length distributions under thermal scenarios

We projected the potential shift in the size distribution of the individuals available to the fishery under future projected thermal scenarios and observed extreme events using the 2003 MHW above described. These projections were compared to the current climate conditions. The hatch-date distribution obtained from the spawning probability model was therefore used to set the individual birthdates of a simulated dolphinfish population (10^6^ individuals) in each of the scenarios (Fig. 4).

For each scenario, the individual growth trajectories were independently estimated for each simulated fish according to the fish-specific daily temperature and photoperiod at birth (which were determined not only by the temperature scenario but also by the fish-specific hatch date), using the growth model described in this study and incorporating the ecological and fishery drivers.

Each fish was assumed to experience a constant instantaneous natural mortality rate (*m*). The value for *m* was estimated from the Hoenig_nls_ empirical prediction model using the maximum lifespan (*t*_*max.*_) known for a given species. This was assumed to be the best natural mortality empirical estimator according to a review of more than 200 species^73^. The *t*_*max*_ value for the Mediterranean dolphinfish individuals was 3 years^74^, which yielded an estimated *m* = 1.703 year^−1^ for the Mediterranean Sea. The fishing mortality (*f*) was estimated to be 3–6 year^−1^ ^75^. For simplicity, this uncertainty was ignored and we arbitrarily set *f* = 5 year^−1^. This mortality rate only operated (1) when a given individual had reached the size of vulnerability to fishing, which was set at 20 cm, as this was the approximate average FL of the minimum size at catch in the available time series, and (2) once the fishery is open. The opening of the fishing season was set as August 15th, which is the opening date of the fishery established in the Mediterranean Sea (Recommendation GFCM/30/2006/2), and we calculated the distribution of the sizes one month later, which coincided with the seasonal peak of catches (evaluation date: September 15th). The probability that a given fish (*i*) had survived from birth until the evaluation date (*P*_*i*_) is given by:

4 $${P}_{i}={e}^{\left(-{m}_{age}-{f}_{days.vulnerable}\right)}$$

The survival until the evaluation date of a simulated fish was evaluated by comparing *P*_*i*_ with a random number between zero and one.

We projected the length distribution of the fish surviving at least to the evaluation date for all thermal scenarios. The R script used to implement these individual-based simulations, together with the technicalities related to uncertainty propagation, are provided as Supplementary Information.

## Supplementary Information

Supplementary Information.
