# Supplementary material for: Projected effects of ocean warming on an iconic pelagic fish and its fishery
Source: Sci Rep. 2021 Apr 22;11:8803. doi: 10.1038/s41598-021-88171-1 (PMC8062520; doi:10.1038/s41598-021-88171-1)
Supplement: Supplementary file 1 — Supplementary Information. [file 41598_2021_88171_MOESM1_ESM.docx]

Supplementary information for the manuscript:

**Projected effects of ocean warming on an iconic pelagic fish and its fishery.**

Vicenç Moltó, Miquel Palmer, Andrés Ospina-Álvarez, Sílvia Pérez-Mayol, Amina Besbes Benseddik, Mark Gatt, Beatriz Morales-Nin, Francisco Alemany, Ignacio A. Catalán

Contents:

1. Figure S1. Map of the study area showing the contrasting SST thermal regimes in the sampling regions.
2. Description of the datasets used to set up, calibrate, and validate de biochronology.
3. Description of the otolith processing and reading.
4. Figure S2. Detail of the otolith section and daily growth increments counts on the dorsal axis.
5. Additional information of the Oceanographic/Climatic models used to obtain the future thermal scenarios to perform the length at catch simulations.
6. Extended equations of the growth models.
7. Table S1. Summary of the parameter values estimated with the growth model.
8. Figure S3. Thermal scenarios used in growth projections.
9. The R data and scripts of the models developed in this study. Available in the public domain <https://figshare.com/account/home> under Creative Commons by 4.0 license https://doi.org/10.6084/m9.figshare.13095638.v1.
10. Figure S1. Map of the study area with polygons over the regions sampled and where daily average SST values were derived to construct the otolith biochronology. Examples of SST correspond to the spawning conditions on July 15, 2007. Temperature data resolution of 0.0417x0.0417 degrees, Myoceans product SST_MED_SST_L4_REP_OBSERVATIONS_010_021. Latitudinal and longitudinal boundaries for the areas and approximate size (squared nautical miles, excluding land) are: Balearic Islands approx. size= 12000 nm^2^. N =40°30’N, S=38°45’N, E =4°12’E, W= 1°59’E; Tunisia approx. size= 12700 nm^2^; N=37°50’N, S=35°30’N, E=11°35’E, W=8°40’E. Malta approx. size=19600 nm^2^ nm^2^; N=36°37’N, S=34°45’N, E=15°45’E, W=13°2’E. Sicily approx. size= 19800 nm^2^; N=39°0’N, S=36°30’N, E=12°30’E, W=16°0’E. Created with R 3.6.2^1^ using the ggmap package^2^.


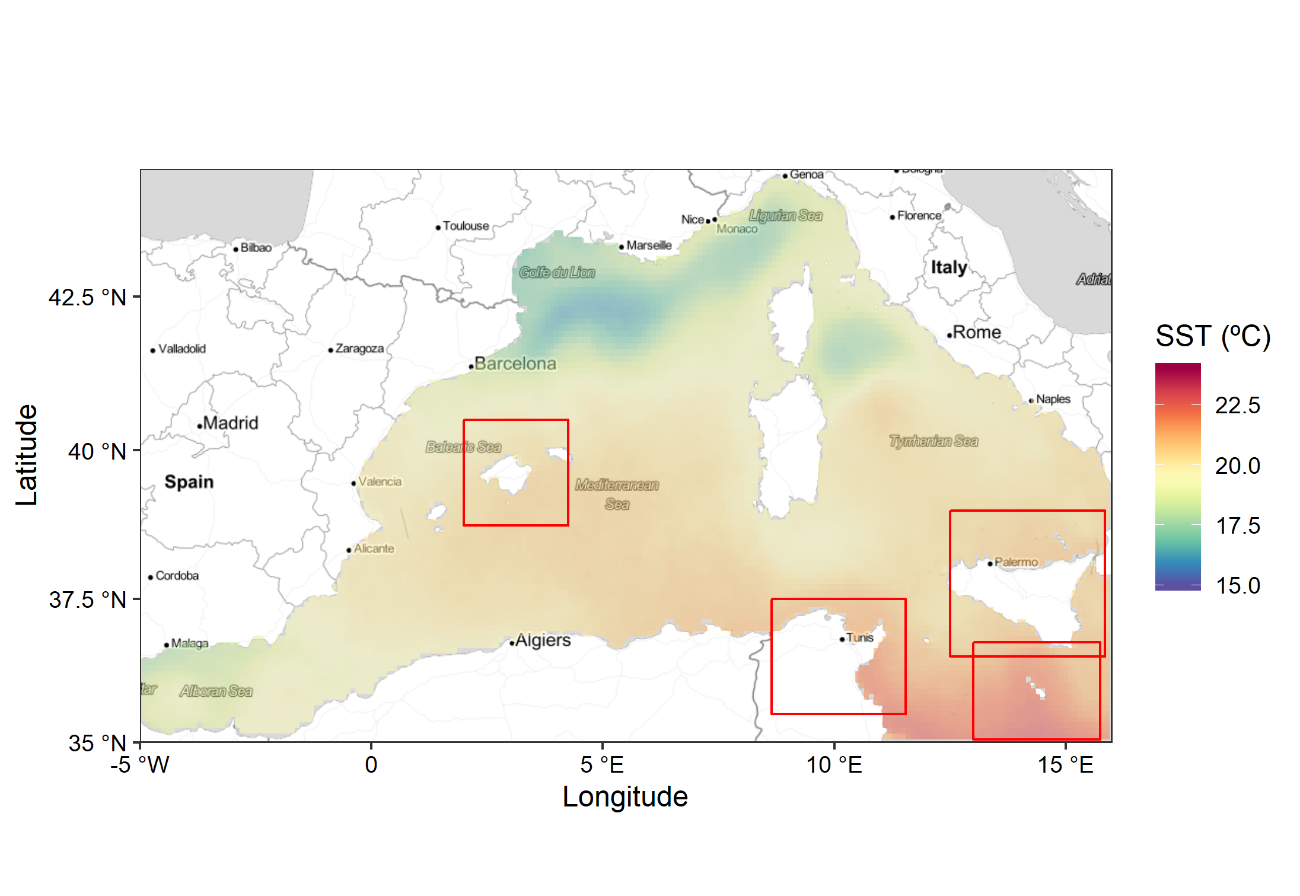


1. Description of the datasets used to set up, calibrate, and validate de biochronology.

The individuals selected for the juvenile otolith readings were obtained from commercial landings of the FAD fishery from Mallorca Island (northwestern Mediterranean) and Tunisia (southern central Mediterranean), which were the areas that provided the most contrasting thermal values for our samples. The furcal length (FL) and capture date were known for each individual. The maximum length was limited to 65 cm FL, which is the maximum length from which juvenile sagittal otoliths can be safely read in this species^3^. After applying strongly restrictive criteria after the otolith reading, according to which only otoliths with clearly visible daily increments and with 100% agreement between two independent readers were used, only 20 individuals were selected to build the initial otolith dataset. The ages of these individuals were between 53 and 135 days old, with lengths ranging from 27 to 55.2 cm FL, and the individuals were born from June to August (earlier and later within the spawning season). This otolith reading dataset resulted in 1894 daily increment widths.

The model was calibrated and validated with a different dataset than the one used to estimate the model parameters (otolith readings). This calibration and validation dataset comprised 1876 age-0 individuals, for which the FL at catch (limited to 65 cm FL), age (total days after hatch), capture date, and spatial origin (boxes in the Fig. S1) were known. These data were obtained from past research projects within these Mediterranean regions during the periods from 1990-1991, 1995-1997, and 2004-2013. This dataset was randomly split into a calibration dataset (1000 individuals) to calibrate the growth pattern from the otolith radius to the FL scale and a validation dataset (the remaining 876 individuals) to validate the accuracy and precision of the FL predicted by the model.

1. Description of the otolith processing and reading.

Saggitae otoliths, either left or right indistinctly, were extracted and processed according to other recent works in which transversal sections have been used^4–7^ to determine the width of their daily rings. The dry and clean otoliths were embedded in epoxy resin, dried for 24h and cut in transversal sections, containing the core of the otolith, using a low-speed diamond saw. The sections were mounted on a microscope glass slide, fixed with thermoplastic glue (Crystalbond®), and sanded with 20µm, 15µm, and 3µm grit sandpaper progressively until the nucleus zone was visible. Then, the glue was reheated, the polished side glued facing down, and the sanding process was repeated. The latter process was repeated until the nucleus was visible. Finally, the surface was further polished with 0.3µm aluminum oxide powder suspension until the nucleus, and the daily growth increments were clearly visible. The sections were photographed at 200X with a Leica DFC425C camera attached to a Leica DRMA2 microscope. The micro-increments were counted and measured on these images using the Image J software^8^ and verified at 400X by observing directly on the microscope. The reading axis was from the core to the dorsal edge, following the maximum angle between each daily ring. The reference points to determine the distance between daily rings were placed on the center of each ring to smooth the distances between the daily increments counts (Figure S2).

The otoliths that did not have a clear nucleus, or those exhibiting over polished edges, were discarded. Each otolith was read by two researchers, and only the otoliths with a 100% agreement between the two reads were used. We assumed that the first increment deposition starts at the hatching date^9^, and the rings are laid down daily^3,10^, so no correction was necessary to estimate age or date of birth, which was inferred by subtracting the age from the capture date.

1.
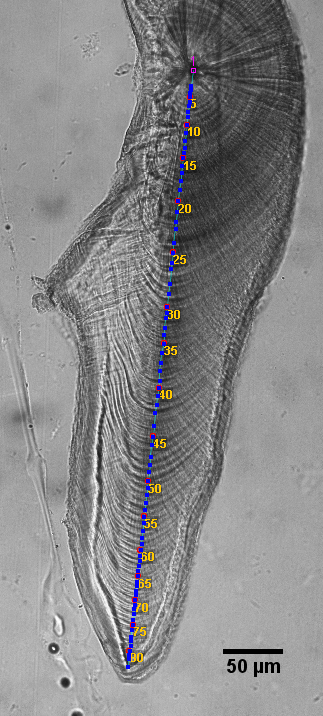
Fig. S2. Detail of the otolith section and daily growth increments counts on the dorsal axis. Photography taken by: Vicenç Moltó.
2. Additional information of the Oceanographic/Climatic models used to obtain the future thermal scenarios to perform the length at catch simulations.

The physical model was the Proudman Oceanographic Laboratory Coastal Ocean Modelling System (POLCOMS; Holt and James, 2001), a 3-D model that can capture the oceanographic dynamics of both the deep ocean and the continental shelf. The model was regionalized for different climate change scenarios in Europe until 2100 within the European project “Climate change and European aquatic resources” (CERES)^12^ and has been shown to reasonably reproduce the main 3D seasonal dynamics in the study area^13^.

The greenhouse gas scenarios (RCP 4.5 and RCP 8.5 from IPCC) were obtained from the data from the global climate model MPI-ESM-LR (<http://www.mpimet.mpg.de/en/science/models/mpi-esm.html>), which were downscaled (to approximately 11 km) to the regional climate model. RCP 4.5 and RCP 8.5 represent moderate and high greenhouse gas concentrations that would increase radiative forcing on Earth by 4.5 W m^-2^ and 8.5 W m^-2^, respectively, by the end of the 21st century^14^. Our projections fell within the intermediate trajectories of global ensemble models for the area in each scenario, and thus, they were considered proxies of potential scenarios of warming in the NW Mediterranean^12^.

The slight SST overestimation of these models was corrected for each climatological week through a correction factor derived from comparing the observed (satellite SST, using the CMEMS product described above) and the SST modeled during the current conditions period. The weekly correction factor (mean = 0.3°C) was applied to future scenarios, for which weekly the thermal means and standard deviations were derived.

1. Extended equations of the growth models.

Equation 1:

$${OtR}_{\left( i,age \right)}=L_{\infty\left( i,age \right)}e^{-b_{\left( i \right)}^{e^{\left( -c_{\left( i \right)}*age \right)}}}+{Rho}_{1}\left( {OtR}_{\left( i,age-1 \right)}-{OtR}_{\left( i,age-1 \right)}^{observed} \right)+{Rho}_{2}\left( {OtR}_{\left( i,age-2 \right)}-{OtR}_{\left( i,age-2 \right)}^{observed} \right)$$

Equation 2:

$$L_{\infty\left( i,age \right)}=L_{\infty,0\left( i \right)}+L_{\infty,slope}{CummTemperature}_{age}$$

Equation 3:

$${FL}_{\left( i \right)}={OtR}_{(i)}\left( \beta_{0}+\beta_{T}T_{b\left( i \right)}+\beta_{P}P_{b\left( i \right)} \right)$$

1. Table S1. Summary of the parameter values estimated with the growth model. The confidence intervals are represented by the 2.50%, 50% (median) and 97.5% values. The other descriptors are the potential scale reduction statistic (R hat), and the effective sample size (N eff).

| **Parameters** | **2.50%** | **50%** | **97.50%** | **R hat** | **N eff** |
| --- | --- | --- | --- | --- | --- |
| L_∞_ mean | 166.055 | 194.755 | 234.746 | 1.00 | 601 |
| L_∞_ sd | 58.181 | 92.224 | 99.713 | 1.00 | 920 |
| L_∞_ slope | 0.076 | 0.087 | 0.099 | 1.01 | 666 |
| b mean | 3.154 | 3.271 | 3.385 | 1.00 | 5545 |
| b sd | 0.160 | 0.225 | 0.337 | 1.00 | 29210 |
| c mean | 0.061 | 0.065 | 0.069 | 1.01 | 4722 |
| c sd | 0.006 | 0.008 | 0.013 | 1.00 | 22538 |
| *β_0_* | 0.187 | 0.222 | 0.261 | 1.01 | 490 |
| *β_T_* | 0.001 | 0.002 | 0.002 | 1.00 | 655 |
| *β_P_* | 0.000 | 0.000 | 0.000 | 1.01 | 562 |
| *Rho1** | 1.450 | 1.498 | 1.546 | 1.00 | 16533 |
| *Rho2** | -0.540 | -0.491 | -0.442 | 1.00 | 18655 |

* Rho are the parameters of the temporal autocorrelation terms. Rho1 and 2 refer to the first and second lag terms, respectively.

1.
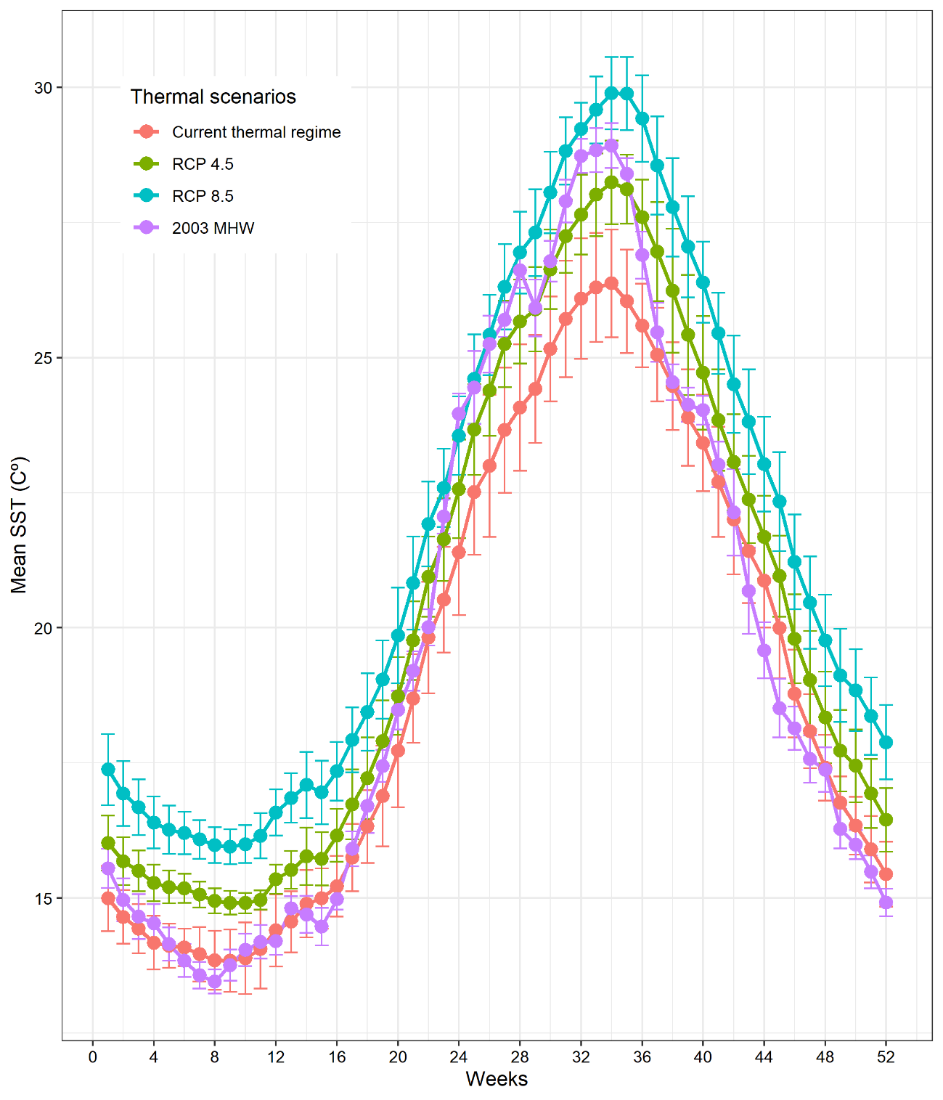
Figure S3. Thermal scenarios used in growth projections built around the Balearic, calculated from the box in Figure S1. The “current thermal regime” (1995-2005) is based on model outputs corrected from satellite-derived observations for the period, averaged over pixels at the same scales as RCP models (11 km pixels). RCP scenarios are projected for the period 2080-2099. MHW 2003 corresponds to the Marine Heat Wave reported in the Mediterranean in 2003 (see methods), over the same area, and at the same resolution. Temperature values for the MHW are satellite data because the model does not correctly reproduce heatwaves. Error bars correspond to the standard deviations of each weekly average. For the MHW 2003, mean and sd. are derived from pixel observations over the integrated area (see methods). Created with R 3.6.2^1^ using the ggplot2 package^15^.

REFERENCES

1. R Core Team. *R: A Language and Environment for Statistical Computing. Version 3.6.2.* https://www.r-project.org/ (R Fundation for Statistical Computing, 2019).

2. Kahle, D. & Wickham, H. ggmap: Spatial Visualization with ggplot2. *R J.* **5**, 144–161 (2013).

3. Massutí, E., Morales-Nin, B. & Moranta, J. Otolith microstructure, age, and growth patterns of dolphin, *Coryphaena hippurus*, in the western Mediterranean. *Fish. Bull.* **97**, 891–899 (1999).

4. Benseddik, A. B. *et al.* Determination of age and growth of dolphinfish, *Coryphaena hippurus*, off Tunisia by otolith microstructure analysis. *Cybium* **35**, 173–180 (2011).

5. Gatt, M., Dimech, M. & Schembri, P. J. Age, Growth and Reproduction of *Coryphaena hippurus* (Linnaeus, 1758) in Maltese Waters, Central Mediterranean. *Mediterr. Mar. Sci.* **16**, 334–345 (2015).

6. Lessa, R. & Santana, F. M. Growth of the dolphinfish *Coryphaena hippurus* from north-eastern Brazil with an appraisal of the efficacy of scales and otoliths for ageing. *J. Fish Biol.* **89**, 977–989 (2016).

7. Schwenke, K. L. & Buckel, J. A. Age, growth, and reproduction of dolphinfish (*Coryphaena hippurus*) caught off the coast of North Carolina. *Fish. Bull.* **106**, 82–92 (2008).

8. Schneider, C. A., Rasband, W. S. & Eliceiri, K. W. NIH Image to ImageJ: 25 years of image analysis. *Nat. Methods* **9**, 671 (2012).

9. Uchiyama, J. H., Burch, R. K. & Kraul, S. A. Growth of dolphins, *Coryphaena hippurus* and *C. equiselis*, in hawaiian waters as determined by daily increments on otoliths. *Fish. Bull.* **84**, 186–191 (1986).

10. Oxenford, H. A. & Hunte, W. Age and growth of dolphin, *Coryphaena hippurus*, as determined by growth rings in otoliths. *Fish. Bull.* **84**, 906–909. (1983).

11. Holt, J. T. & James, I. D. An s coordinate density evolving model of the northwest European continental shelf 1, Model description and density structure. *J. Geophys. Res.* **106**, 14015–14034 (2001).

12. Peck, M. A. *et al.* *Climate change and European Fisheries and Aquaculture: ‘CERES’ Project Synthesis Report.* (2020) doi:10.25592/uhhfdm.804.

13. Ramírez-Romero, E. *et al.* Assessment of the Skill of Coupled Physical-Biogeochemical Models in the NW Mediterranean. *Front. Mar. Sci.* (2020).

14. Alexander, M. A. *et al.* Projected sea surface temperatures over the 21st century: Changes in the mean, variability and extremes for large marine ecosystem regions of Northern Oceans. *Elem. Sci. Anthr.* **6**, 25 (2018).

15. Wickham, H. ggplot2. *Wiley Interdiscip. Rev. Comput. Stat.* **3**, 180–185 (2011).
